# Association Between Prolonged Immobilization and Venous Thromboembolism in Hospitalized Medical Patients

**DOI:** 10.7759/cureus.111189

**Published:** 2026-06-20

**Authors:** Jamil Ahmad, Syed Muhammad Ali Shah, Haider Imran, Iffat Siddiq, Dure Nayab, Nadir Imran, Shifa Haleem, Tabish Sattar, Sumaria Nazir, Mahwash Nazir, Sundas Safdar

**Affiliations:** 1 Internal Medicine, Lady Reading Hospital, Peshawar, PAK; 2 Geriatrics, Naas General Hospital, Naas, IRL; 3 Internal Medicine, Foundation University Medical College, Islamabad, PAK; 4 Medicine, Saidu Group of Teaching Hospitals, Saidu Sharif, PAK; 5 Medicine, Samarkand State Medical University, Samarkand, UZB; 6 Medicine, Hayatabad Medical Complex, Peshawar, PAK; 7 Public Health, Cardiff University, Cardiff, GBR; 8 Medicine, Borders General Hospital, Melrose, GBR; 9 Diagnostic Radiology, Lady Reading Hospital, Peshawar, PAK

**Keywords:** deep vein thrombosis, hospitalized medical patients, prolonged immobilization, pulmonary embolism, venous thromboembolism

## Abstract

Background

Venous thromboembolism (VTE) is a potentially life-threatening but preventable complication among hospitalized medical patients.

Objective

To evaluate the association between the duration of immobilization and the risk of VTE (deep vein thrombosis and/or pulmonary embolism) in hospitalized medical patients.

Methodology

The analytical observational study was conducted at two tertiary care hospitals in Peshawar, namely, Lady Reading Hospital and Hayatabad Medical Complex, from January 2025 to December 2025. Consecutive sampling of 410 hospitalized medical patients ≥18 years of age resulted in the enrollment of all patients. Patients were excluded if they had a prior history of VTE and/or therapeutic anticoagulation. Immobilization was categorized as mild (<24 hours of restricted movement), moderate (24-72 hours of reduced mobility), and prolonged (>72 hours of complete or near-complete bed rest). Diagnosis of VTE was made with Doppler ultrasonography and computed tomography (CT) pulmonary angiography. The data were analyzed using SPSS version 26 (IBM Corp., Armonk, NY). A chi-square test and multivariable logistic regression were performed, and a p-value of ≤ 0.05 was deemed to be significant.

Results

Among 410 patients, VTE occurred in 68 (16.59%) cases, while 342 (83.41%) remained free of VTE. The incidence of VTE was higher for patients with increased immobilization, occurring in 8/146 (5.48%) patients with mild immobilization, 22/162 (13.58%) with moderate immobilization, and 38/102 (37.25%) with prolonged immobilization (p < 0.001). Prolonged immobilization was the strongest independent predictor of VTE with an adjusted odds ratio of 4.68 (95% CI: 2.72-8.05).

Conclusion

Prolonged immobilization is independently associated with increased odds of VTE among hospitalized medical patients.

## Introduction

Venous thromboembolism (VTE) is one of the leading and preventable causes of morbidity and mortality in inpatient settings [1,2]. It includes deep vein thrombosis (DVT) and pulmonary embolism (PE), which refer to the development of a thrombosis in the venous circulation that may embolize into the pulmonary circulation [3]. VTE continues to be a major health problem in the world, especially for hospitalized patients, who often have several acquired risk factors that combine to enhance their thrombotic risks [4].

One of the most significant and well-known modifiable risk factors for VTE is prolonged immobility [5]. It ultimately results in venous stasis, along with endothelial injury and hypercoagulability [6], all of which are part of Virchow's triad. In hospitalized patients, prolonged immobilization was defined as either complete bed rest or markedly reduced mobility for ≥3 consecutive days, or functional limitation resulting in less than two hours of ambulation or purposeful movement per day while still requiring bed rest [7]. Under these circumstances, calf muscle pump activity plays an important role in returning venous blood from the lower limbs. Loss of this mechanism leads to increased venous pooling and thrombus formation. Furthermore, immobilization is often accompanied by systemic inflammation and even dehydration, along with increased severity of acute illness, all of which further contribute to a prothrombotic state [8].

Hospitalized medical patients are at high risk of VTE due to multiple overlapping risk factors, including advanced age, acute infection, heart failure, stroke, malignancy, and metabolic comorbidities [9,10]. Although pharmacological thromboprophylaxis is recommended in clinical guidelines, VTE still occurs in approximately 1-2% of hospitalized medical patients even when prophylaxis is appropriately administered, with higher rates observed in high-risk subgroups such as patients with cancer and critically ill patients [11-13]. Furthermore, a substantial proportion of hospital-associated VTE events are diagnosed after discharge or become clinically evident only when complications arise, highlighting immobilization as a dynamic and evolving risk factor rather than a fixed patient characteristic.

The Padua Prediction Score is widely used for VTE risk stratification in hospitalized medical patients, where reduced mobility is incorporated as a key and potentially time-dependent component [14]. Because both clinical condition and mobility status may change during hospitalization, periodic reassessment of VTE risk is recommended to ensure that prophylaxis decisions remain aligned with the patient’s current risk profile. In addition, the degree and duration of immobilization vary according to disease severity, treatment approach, and recovery trajectory. However, in routine clinical practice, immobilization is often poorly documented and rarely quantified, which complicates accurate risk assessment. This variability underscores the importance of characterizing specific immobilization patterns and their association with thromboembolic outcomes in real-world inpatient settings. The objective of this study was to evaluate the relationship between prolonged immobilization and the development of VTE in medically hospitalized patients.

## Materials and methods

Study design and settings

This was a prospective cohort observational study conducted at two major tertiary care hospitals in Peshawar, namely, Lady Reading Hospital and Hayatabad Medical Complex. Eligible patients were consecutively enrolled at admission and prospectively followed during hospitalization to assess the occurrence of VTE in relation to immobilization and other clinical risk factors. VTE screening was performed only when clinically suspected and was not part of routine screening.

Study duration

The study population was recruited over one year (January 2025 to December 2025). Each enrolled patient was prospectively followed during the entire hospital stay, from admission until discharge or occurrence of VTE, to ensure in-hospital detection of study outcomes.

Inclusion and exclusion criteria

Patients aged ≥18 years admitted to the medical wards were included. Only those with a hospital stay of at least 48 hours were eligible for participation. To minimize confounding and improve internal validity, patients with active pre-existing VTE who were receiving therapeutic anticoagulation prior to admission were excluded, along with pregnant patients and those with known inherited thrombophilia. Patients with a previous history of VTE who were not on anticoagulation at admission were included, as their inclusion allowed evaluation of current immobilization-related risk independent of past events.

Sample size and sampling technique

A total of 410 patients were included in the study using non-probability consecutive sampling, whereby all eligible patients admitted during the study period were enrolled sequentially until the target sample size was reached. This approach ensured representation of routine hospital admissions while minimizing selection bias. A formal sample size calculation was not performed; rather, all eligible patients were consecutively included during the study period until the predefined sample size of 410 was achieved.

Data collection

Data were collected using a structured proforma developed by the hospital research team, comprising consultant physicians and resident doctors, after obtaining informed consent from patients or their attendants. The proforma was designed to ensure standardized and uniform documentation of all study variables, including demographic characteristics, medical history, comorbid conditions, duration and severity of immobilization, VTE risk factors, laboratory parameters, and imaging findings. To ensure clarity, feasibility, and completeness of the tool, the proforma was expert-reviewed by senior clinicians and subsequently pilot-tested on 20 patients prior to formal data collection. This pilot phase was used to identify and resolve any issues related to wording, structure, and practical applicability, after which minor refinements were incorporated into the final version.

Clinical data were recorded at the time of hospital admission, and patients were prospectively followed throughout their hospital stay for the development of clinical features suggestive of VTE. In suspected cases, appropriate diagnostic investigations were arranged, and only radiologically confirmed cases (compression Doppler ultrasonography for DVT and CT pulmonary angiography for PE) were considered as outcome events. Severity of acute illness was categorized clinically by the treating physician as mild, moderate, or severe based on overall clinical condition, vital stability, and requirement of intensive monitoring or ICU admission.

Variables of the study

Independent variables included the degree and duration of immobilization, age, gender, presence of diabetes mellitus, hypertension, heart failure, acute infection, stroke, and severity of acute medical illness. Immobilization was operationally categorized as mild, moderate, and prolonged based on the level and duration of ambulation during hospitalization. Mild immobilization referred to reduced mobility with preserved ability to ambulate for ≥2-3 hours per day, moderate immobilization referred to limited mobility with ≤2 hours of daily ambulation and predominantly bed-to-chair activity, while prolonged immobilization was defined as complete bed rest or minimal movement (≤1 hour of ambulation per day) for ≥3 consecutive days. These definitions were adapted from established clinical guidelines on VTE prevention in hospitalized medical patients [15]. Immobilization classification was applied at admission and updated during hospitalization when clinically relevant changes in mobility status occurred.

The dependent variable was VTE, defined as radiologically confirmed DVT and/or PE diagnosed during hospitalization. DVT was diagnosed by compression Doppler ultrasonography demonstrating non-compressibility of the deep veins of the lower limbs, while PE was confirmed by CT pulmonary angiography showing intraluminal filling defects in the pulmonary arteries. Only imaging-confirmed cases were considered as outcome events. Confounding variables included obesity, smoking, malignancy, dehydration, use of central venous catheters, and medications affecting coagulation, such as steroids and hormonal therapy.

Statistical analysis

SPSS version 26 (IBM Corp., Armonk, NY) was used for data analysis. All data were expressed in terms of mean and standard deviations for continuous variables and frequencies and percentages for the categorical variables. The relationship between prolonged immobilization and VTE was evaluated using the chi-square test. Multivariable logistic regression was used to adjust for potential VTE confounders and identify independent predictors of VTE; results are presented as adjusted odds ratios (AORs) and 95% confidence intervals. The multivariable logistic regression model included immobilization category, age, gender, diabetes mellitus, hypertension, heart failure, stroke, acute infection, obesity, malignancy, dehydration, central venous catheter use, smoking, and medication use (steroids and hormonal therapy). Data regarding pharmacological or mechanical thromboprophylaxis during hospitalization were not systematically recorded and therefore were not included in the analysis. P-values ≤0.05 were deemed statistically significant.

Ethical considerations

Ethical approval was obtained from the Institutional Review Board (IRB) of Lady Reading Hospital Peshawar before the study was started. All participants/legal guardians signed informed consent forms. Patient confidentiality was respected, and data were only used for research purposes and not for providing any identifiable personal information.

## Results

A total of 410 hospitalized medical patients were included in the study (Table 1). The majority of patients were aged 41-60 years (172, 41.95%), followed by >60 years (140, 34.15%), while 98 (23.90%) were aged 18-40 years. Males constituted 232 (56.59%) of the study population, whereas females accounted for 178 (43.41%). Among comorbid conditions, hypertension was present in 168 (40.98%) patients, diabetes mellitus in 142 (34.63%), and acute infection in 154 (37.56%). Heart failure was observed in 76 (18.54%), stroke in 88 (21.46%), and smoking history was noted in 119 (29.02%) patients. Additional risk factors included obesity in 96 (23.41%), dehydration in 102 (24.88%), malignancy in 54 (13.17%), central venous catheter use in 73 (17.80%), steroid therapy in 119 (29.02%), and hormonal therapy in 41 (10.00%).

**Table 1:** Baseline demographic characteristics and clinical risk factors of the study population (n = 410).

Variable	Category	Frequency and percentage, n (%)
Age (years)	18-40	98 (23.90%)
	41-60	172 (41.95%)
	>60	140 (34.15%)
Gender	Male	232 (56.59%)
	Female	178 (43.41%)
Diabetes mellitus	Yes	142 (34.63%)
Hypertension	Yes	168 (40.98%)
Heart failure	Yes	76 (18.54%)
Acute infection	Yes	154 (37.56%)
Stroke	Yes	88 (21.46%)
Smoking	Yes	119 (29.02%)
Obesity	Yes	96 (23.41%)
Malignancy	Yes	54 (13.17%)
Dehydration	Yes	102 (24.88%)
Central venous catheter	Yes	73 (17.80%)
Steroid therapy	Yes	119 (29.02%)
Hormonal therapy	Yes	41 (10.00%)

**Figure 1:**
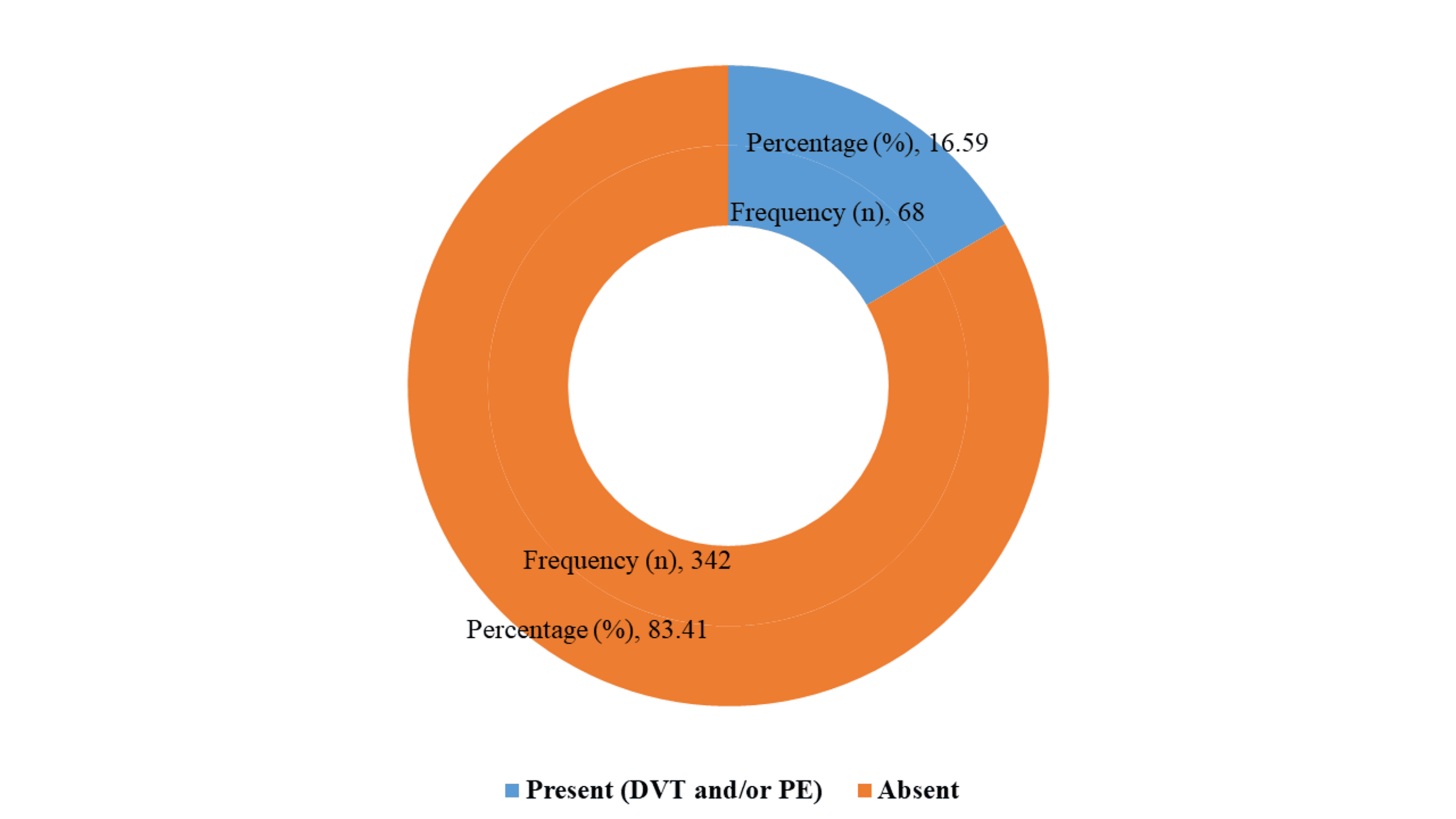
VTE status in the study population (N = 410). DVT: deep vein thrombosis; PE: pulmonary embolism.

Out of 410 patients, VTE, including DVT, PE, or both, was diagnosed in 68 patients, yielding an overall incidence of 16.59%. The remaining 342 patients (83.41%) did not develop VTE during hospitalization (Figure 1).

A significant association was observed between immobilization status and the occurrence of VTE (p < 0.001), as shown in Table 2. VTE incidence increased progressively with worsening immobilization. Patients with mild immobilization had a VTE rate of 5.48% (8/146), which increased to 13.58% (22/162) in moderately immobilized patients. The highest incidence was observed in patients with prolonged immobilization, where 37.25% (38/102) developed VTE. Overall, chi-square analysis demonstrated a strong statistically significant association (χ² = 48.62, p < 0.001), indicating a clear dose-response relationship between immobilization severity and VTE occurrence.

**Table 2:** Association between immobilization and VTE (chi-square analysis). Immobilization categories were defined based on the degree and duration of reduced mobility during hospitalization, including daily ambulation time and bed-rest status, as described in the Methods section. VTE: venous thromboembolism.

Immobilization	VTE present	VTE absent	Total	VTE rate (%)	P-value	χ² value
Mild	8	138	146	5.48%	<0.001	48.62
Moderate	22	140	162	13.58%		
Prolonged	38	64	102	37.25%		
Total	68	342	410	16.59%		

**Figure 2:**
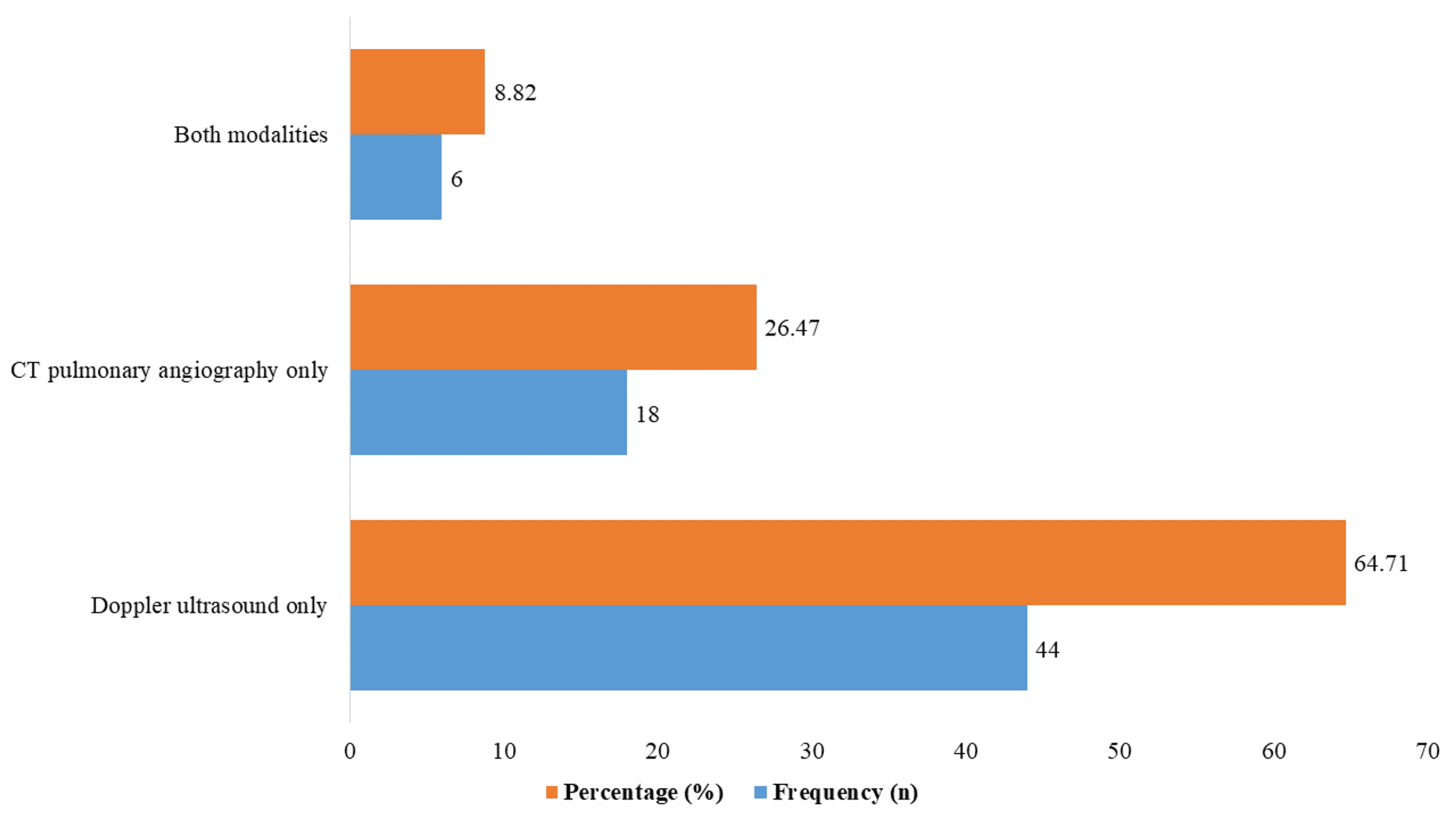
Type of venous thromboembolism (n = 68).

Among 68 patients with VTE, DVT was the most common presentation, observed in 44 patients (64.71%). PE was diagnosed in 18 patients (26.47%), while six patients (8.82%) had both DVT and PE concurrently (Figure 2).

**Figure 3:**
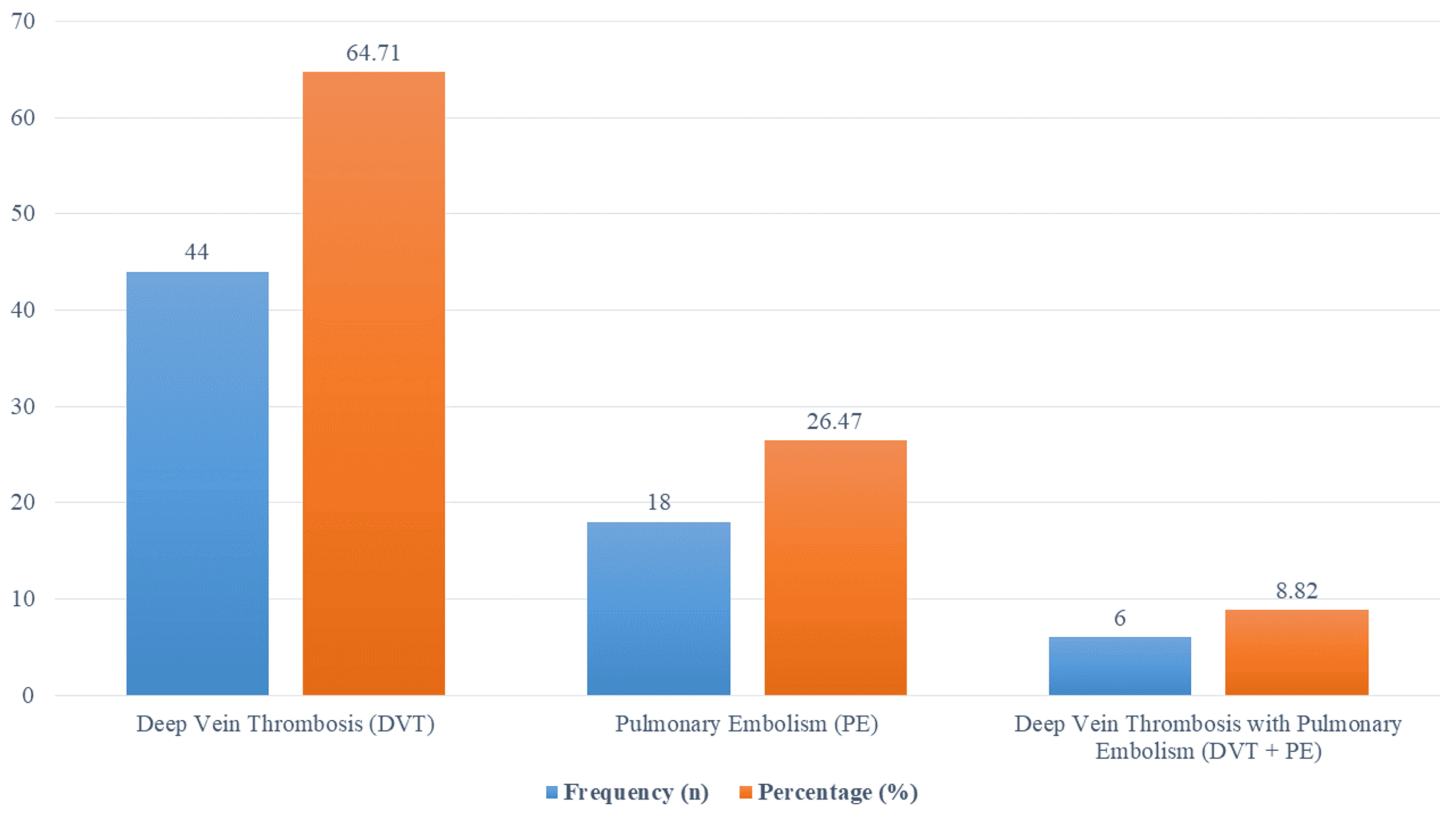
Diagnostic modalities used for the confirmation of venous thromboembolism (n = 68).

Regarding diagnostic confirmation, Doppler ultrasonography alone was used in 44 cases (64.71%), CT pulmonary angiography alone in 18 cases (26.47%), and both imaging modalities were required in six cases (8.82%) for definitive diagnosis of VTE (Figure 3).

Multivariable logistic regression revealed that prolonged immobilization was the strongest independent predictor of VTE (AOR: 4.68, 95% CI: 2.72-8.05, p < 0.001), followed by moderate immobilization (AOR: 2.21, 95% CI: 1.23-3.98, p = 0.008), as shown in Table 3. Other significant predictors included malignancy (AOR: 2.91, p = 0.003), heart failure (AOR: 2.36, p = 0.009), central venous catheter use (AOR: 2.12, p = 0.023), stroke (AOR: 1.88, p = 0.046), obesity (AOR: 1.74, p = 0.044), and age >60 years (AOR: 1.92, p = 0.027). Diabetes mellitus, hypertension, smoking, dehydration, steroid use, and hormonal therapy were not statistically significant predictors of VTE.

**Table 3:** Multivariable logistic regression for independent predictors of VTE. The multivariable logistic regression model included immobilization category, age, diabetes mellitus, hypertension, heart failure, stroke, acute infection, obesity, malignancy, smoking, dehydration, central venous catheter use, steroid therapy, and hormonal therapy. VTE: venous thromboembolism; AOR: adjusted odds ratio.

Variable	AOR	95% CI	p-value
Prolonged immobilization	4.68	2.72-8.05	<0.001
Moderate immobilization	2.21	1.23-3.98	0.008
Age >60 years	1.92	1.08-3.40	0.027
Diabetes mellitus	1.58	0.91-2.74	0.102
Hypertension	1.41	0.82-2.43	0.212
Heart failure	2.36	1.24-4.49	0.009
Stroke	1.88	1.01-3.51	0.046
Acute infection	1.67	0.96-2.90	0.069
Obesity	1.74	1.01-3.02	0.044
Malignancy	2.91	1.45-5.82	0.003
Smoking	1.39	0.81-2.38	0.232
Dehydration	1.63	0.94-2.84	0.081
Central venous catheter	2.12	1.11-4.05	0.023
Steroid therapy	1.48	0.86-2.54	0.154
Hormonal therapy	1.71	0.83-3.53	0.145

## Discussion

The current study showed that prolonged immobilization was strongly associated with VTE in hospitalized medical patients and was statistically significant. The overall incidence of VTE was 16.59% (68/410), with a marked dose-response relationship across immobilization categories: 5.48% (8/146) in mild, 13.58% (22/162) in moderate, and 37.25% (38/102) in prolonged immobilization (χ² = 48.62, p < 0.001). This corroborates the results of a large epidemiological meta-analysis of studies that reported immobilization quadruples the risk of VTE in medical patients (RR: 1.86, 95% CI: 1.61-2.14), suggesting that venous stasis is important as a mechanistic component to the development of VTE [16].

Among the multiple variables, prolonged immobilization was the strongest independent factor associated with VTE in the present study (p < 0.001, AOR: 4.68; 95% CI: 2.72-8.05), whereas moderate immobilization was also significantly associated (AOR: 2.21, p = 0.008). A similar case-crossover study showed that immobilization was associated with an almost 20-fold increased risk for VTE (OR: 19.8, 95% CI: 11.5-34.0) [17]. The less pronounced effect in our study might be due to differences in case definition, heterogeneity of populations, and prophylaxis.

The incidence of VTE in subjects who were immobilized for a long time in our study was 37.25%, which is significantly greater than the incidence of VTE mentioned in many reports from other countries. In fact, a systematic review of temporary lower limb immobilization found that absolute rates of VTE were much lower but still clinically relevant, especially in orthopedic trauma patients [18]. The difference might be due to the inclusion of acutely ill medical inpatients with systemic inflammation, dehydration, and multi-organ dysfunction in addition to immobility, which may increase the thrombogenic potential.

There is also randomized evidence that immobilized hospitalized patients are high-risk patients for whom thromboprophylaxis is indicated. It was concluded from a network meta-analysis that pharmacological prophylaxis was effective in reducing VTE incidence in immobilized patients and indicates the clinical significance of the severity of immobilization as a risk stratification factor [19]. This helps to interpret that immobilization is not just a passive risk marker but a therapeutic target in inpatient care.

Furthermore, hospitalization is a strong risk factor for VTE, and immobilization increases this effect. A population-based case-crossover study reported that hospitalization with immobilization increased VTE risk almost 20-fold compared to baseline periods (OR: 19.8, 95% CI: 11.5-34.0) [17]. In contrast to that, our study shows that immobilization is also an independent predictor of the occurrence of a thrombus even after controlling for other factors associated with hospitalization (heart failure, AOR: 2.36; malignancy, AOR: 2.91), suggesting that immobilization plays a major role in the formation of a thrombus.

Finally, other significant predictors in our study included malignancy (AOR: 2.91, p = 0.003), heart failure (AOR: 2.36, p = 0.009), central venous catheter use (AOR: 2.12, p = 0.023), and stroke (AOR: 1.88, p = 0.046). These results corroborate the previously mentioned literature that indicates these are synergistic factors of hypercoagulability and endothelial dysfunction [9,20,21]. However, the most prominent and modifiable risk factor was immobilization, which is indicative of its key role in the pathogenesis of VTE in the inpatient setting.

Study strengths and limitations

This study has several strengths that enhance the validity and applicability of its findings. It was conducted as a prospective observational study in two major, high-volume tertiary care hospitals in Peshawar, which serve as referral centers receiving patients from across the region and other parts of Pakistan. This setting improves the external validity and ensures that the study population reflects a broad spectrum of real-world hospitalized medical patients. The relatively large sample size of 410 patients, along with consecutive sampling, minimized selection bias. In addition, the use of standardized data collection tools, prospective monitoring for clinically suspected VTE, and objective radiological confirmation using compression Doppler ultrasonography and CT pulmonary angiography strengthened diagnostic accuracy and reduced misclassification bias. Furthermore, multivariable logistic regression allowed adjustment for multiple confounders, improving the robustness of the association between immobilization and VTE.

Despite these strengths, several limitations should be acknowledged. Although the study was conducted in major referral hospitals, it remains hospital-based and may not be fully generalizable to community or non-hospitalized populations. Residual confounding may still exist due to unmeasured variables, including genetic thrombophilia and lack of systematically recorded data on pharmacological or mechanical thromboprophylaxis. In addition, although immobilization was classified using predefined criteria, some degree of clinical judgment may have introduced inter-observer variability. Finally, follow-up was limited to the in-hospital period, and no post-discharge surveillance was performed, which may have resulted in underestimation of delayed thromboembolic events occurring after discharge.

## Conclusions

In hospitalized medical patients, prolonged immobilization is significantly associated with an increased risk of VTE. The risk demonstrates a clear upward trend with increasing severity of immobilization, and this association remains significant after adjustment for major comorbid and clinical risk factors. These findings suggest that immobilization is an important and potentially modifiable risk marker that should be identified early during hospitalization, and that timely mobilization strategies along with appropriate thromboprophylaxis may help reduce the burden of hospital-associated VTE. However, given the observational design, the findings should be interpreted as an association rather than evidence of causation.

## Appendices

**Table 4:** Data collection proforma for VTE and immobilization study. VTE: venous thromboembolism; DVT: deep vein thrombosis; PE: pulmonary embolism; CDUS: compression Doppler ultrasonography; CTPA: computed tomography pulmonary angiography; LRH: Lady Reading Hospital; HMC: Hayatabad Medical Complex; CBC: complete blood count.

Section	Variable	Response options/Measurement
A. Patient identification	Study ID	
	Hospital	LRH/HMC
	Date of admission	DD/MM/YYYY
	Age	___ years
	Gender	Male/Female
B. Clinical history	Diabetes mellitus	Yes/No
	Hypertension	Yes/No
	Heart failure	Yes/No
	Stroke	Yes/No
	Acute infection	Yes/No
	Previous history of VTE	Yes/No
	Smoking status	Yes/No
C. Comorbid & risk factors	Obesity	Yes/No (BMI ≥30 kg/m² if available)
	Malignancy	Yes/No
	Dehydration (clinical diagnosis)	Yes/No
	Central venous catheter	Yes/No
	Steroid therapy	Yes/No
	Hormonal therapy	Yes/No
D. Immobilization assessment	Degree of immobilization	Mild/Moderate/Prolonged
	Duration of reduced mobility	<3 days/3-7 days/>7 days
	Bed rest status	Complete/Partial/Ambulatory
	Daily mobility level	Normal/Reduced (<2 hours ambulation)/None
E. Clinical severity indicators	Severity of acute illness	Mild/Moderate/Severe (clinical judgment)
	ICU admission	Yes/No
F. Laboratory & supportive data	Relevant labs recorded	CBC/D-dimer/others if available
G. VTE outcome assessment	Clinical suspicion of VTE	Yes/No
	DVT confirmed by CDUS	Yes/No
	PE confirmed by CTPA	Yes/No
	Type of VTE	DVT/PE/Both
H. Timing of outcome	Time of VTE occurrence	During hospitalization/Not occurred
I. Follow-up status	Outcome at discharge	VTE present/VTE absent
Note/Remarks:		
